# Genetic Analysis Implicates Dysregulation of *SHANK2* in Renal Cell Carcinoma Progression

**DOI:** 10.3390/ijerph191912471

**Published:** 2022-09-30

**Authors:** Chi-Fen Chang, Shu-Pin Huang, Yu-Mei Hsueh, Jiun-Hung Geng, Chao-Yuan Huang, Bo-Ying Bao

**Affiliations:** 1 Department of Anatomy, School of Medicine, China Medical University, Taichung 406, Taiwan; 2 Department of Urology, Kaohsiung Medical University Hospital, Kaohsiung 807, Taiwan; 3 Graduate Institute of Clinical Medicine, College of Medicine, Kaohsiung Medical University, Kaohsiung 807, Taiwan; 4 Department of Urology, Faculty of Medicine, College of Medicine, Kaohsiung Medical University, Kaohsiung 807, Taiwan; 5 Ph.D. Program in Environmental and Occupational Medicine, College of Medicine, Kaohsiung Medical University, Kaohsiung 807, Taiwan; 6 Department of Family Medicine, Wan Fang Hospital, Taipei Medical University, Taipei 116, Taiwan; 7 Department of Public Health, School of Medicine, College of Medicine, Taipei Medical University, Taipei 110, Taiwan; 8 Department of Urology, Kaohsiung Municipal Hsiao-Kang Hospital, Kaohsiung 812, Taiwan; 9 Department of Urology, National Taiwan University Hospital, College of Medicine, National Taiwan University, Taipei 100, Taiwan; 10 Department of Pharmacy, China Medical University, Taichung 406, Taiwan; 11 Sex Hormone Research Center, China Medical University Hospital, Taichung 404, Taiwan; 12 Department of Nursing, Asia University, Taichung 413, Taiwan

**Keywords:** renal cell carcinoma, single-nucleotide polymorphism, SHANK, risk, survival

## Abstract

SH3 and multiple ankyrin repeat domains (SHANK) is a family of scaffold proteins that were first identified to be involved in balancing synaptic transmission via regulation of intracellular signalling crosstalk and have been linked to various cancers. However, the role of the SHANK genes in renal cell carcinoma (RCC) remains to be elucidated. In this study, we aimed to evaluate whether genetic variants in SHANK family genes affect the risk of RCC and survival of patients. A genetic association study was conducted using logistic regression and Cox regression analyses, followed by the correction for a false discovery rate (FDR), in 630 patients with RCC and controls. A pooled analysis was further performed to summarise the clinical relevance of *SHANK* gene expression in RCC. After adjustment for known risk factors and the FDR, the *SHANK2* rs10792565 T allele was found to be associated with an increased risk of RCC (adjusted odds ratio = 1.79, 95% confidence interval = 1.32–2.44, *p* = 1.96 × 10^−4^, *q* = 0.030), whereas no significant association was found with RCC survival. A pooled analysis of 19 independent studies, comprising 1509 RCC and 414 adjacent normal tissues, showed that the expression of *SHANK2* was significantly lower in RCC than in normal tissues (*p* < 0.001). Furthermore, low expression of *SHANK2* was correlated with an advanced stage and poor prognosis for patients with clear cell and papillary RCC. This study suggests that *SHANK2* rs10792565 is associated with an increased risk of RCC and that *SHANK2* may play a role in RCC progression.

## 1. Introduction

Kidney cancer is a common malignancy, and there were approximately 431,288 new cases and 179,368 deaths worldwide in 2020 [1]. Renal cell carcinoma (RCC), which originates from the epithelium of renal tubules, is the most common form of adult kidney cancer, accounting for 85% of the diagnoses [2]. Because of the lack of diagnostic biomarkers, many patients with RCC are diagnosed at an advanced stage and have a poor prognosis [3]. Thus, finding novel biomarkers is important for RCC detection and monitoring.

Human cancers are thought to be driven by the accumulation of genetic mutations and aberrant gene expression. Recent studies have shown that SH3 and multiple ankyrin repeat domains (SHANK) family genes are linked to cancer [4,5]. The SHANK family currently includes SHANK1, SHANK2, SHANK3, and the SHANK-associated RH domain interactor (SHARPIN), which interacts with the SHANK proteins through the ankyrin repeat domain. SHANKs are members of a new family of scaffold proteins that contain multiple domains, such as ankyrin repeats, PSD-95/Discs Large/ZO-1 domain, SRC homology 3 domain, sterile alpha motif domain, and long proline-rich region, for protein–protein interaction. The SHANK family proteins were first found to be highly expressed in the postsynaptic density of excitatory synapses, but subsequent studies have shown that they are also expressed in various organs and localised in the plasma membrane and nucleus of cells [6]. SHANKs interact with actin regulatory molecules, such as cortactin in growth cones, which suggests that these proteins may play roles in cytoskeletal remodelling and cell migration [7]. Furthermore, SHANK proteins interact as scaffolds with surface receptors to facilitate signalling crosstalk among intracellular pathways [8]. It has been reported that SHANK1 and SHANK3 inhibit breast cancer cell migration and invasion by suppressing integrin activity via sequestration of active Rap1 and R-Ras [5]. Other studies have demonstrated that increased expression of SHANK1 and SHANK3 is involved in the development of several types of cancer, including colon, pancreatic, and lung cancers, and in patient prognosis [9,10]. Genetic studies have suggested that genetic variants in *SHANK* genes may predispose individuals to neuropsychiatric disorders, such as schizophrenia, Alzheimer’s disease, and autism spectrum disorders [11,12]. However, direct evidence linking *SHANK* gene variations to the risk and prognosis of RCC is scarce.

As the most common type of genetic variability, single-nucleotide polymorphisms (SNPs) are considered potential biomarkers for the susceptibility of individuals to disease and may play a role in the personalised treatment strategy [13]. Increasing evidence has demonstrated that genetic variants contribute to the susceptibility of their carriers to RCC, and multiple genetic risk loci have been identified in p53, DNA damage response, and apoptosis pathways in recent genome-wide association studies (GWAS) [14]. However, GWAS require large sample collections and stringent adjustment for multiple testing to avoid false positives. The hypothesis-driven candidate pathway approach is value particularly for studying low allele frequencies, small effect sizes, and limited or unique populations. Given the involvement of the SHANK pathway in tumorigenesis, we comprehensively evaluated the associations of 161 SNPs among SHANK family genes with the risk of RCC and survival in a cohort of 630 patients with RCC and controls from Taiwan. Furthermore, the prognostic value of a candidate gene was assessed via a pooled analysis to support the underlying mechanism of observed associations in RCC.

## 2. Patients and Methods

### 2.1. Study Population and Participant Data Collection

In this study, 312 patients with RCC and 318 healthy controls were recruited from three Taipei city hospitals, namely the National Taiwan University Hospital, Taipei Municipal Wan Fang Hospital, and Taipei Medical University Hospital [15,16]. All patients were newly diagnosed with RCC by clinical and pathological tests. The controls were healthy individuals with no history of cancer and were recruited from the physical examination centres of these hospitals during the same period. Trained personnel used a structured questionnaire to obtain demographic characteristics of the participants, and clinical information was obtained from their medical records. The overall survival was defined as the time between the diagnosis and death from any cause. All of the participants were Taiwanese, and there was no blood relationship between them. This study was approved by the Research Ethics Committee of the National Taiwan University Hospital (9100201527) in accordance with the Good Clinical Practice principles, and written informed consent was obtained from all participants before recruitment.

### 2.2. SNP Selection and Genotyping

Haplotype-tagging SNPs were selected from ±10 kb flanking regions of the SHANK family genes (*SHANK1–3* and *SHARPIN*) with the pairwise linkage disequilibrium threshold of *r*^2^ > 0.8 using the data for Han Chinese from the 1000 Genomes Project [17,18]. Genomic DNA was extracted from whole-blood samples of each participant using the QIAamp DNA Blood Midi Kit (Qiagen, Valencia, CA, USA), and genotyped using Affymetrix Axiom genotyping arrays (Thermo Fisher Scientific, Waltham, MA, USA) at the National Centre for Genome Medicine, Taiwan [19]. Quality control was performed to remove SNPs with a minor allele frequency of <0.03, a genotyping rate of <0.95, and a Hardy–Weinberg equilibrium of <0.01. Finally, 161 SNPs remained for further exploration.

### 2.3. Bioinformatics Analyses

The expression quantitative trait locus (eQTL) analysis for *SHANK2* rs10792565 in normal kidney cortex were obtained from the Genotype Tissue Expression (GTEx) eQTL calculator [20]. HaploReg v4.1 (https://pubs.broadinstitute.org/mammals/haploreg/haploreg.php, accessed on 26 September 2022) was used to annotate potential functions of the SNPs [21]. The Cancer Genome Atlas (TCGA) kidney chromophobe (KICH), kidney renal clear cell carcinoma (KIRC), and kidney renal papillary cell carcinoma (KIRP) datasets [22], the Oncomine [23], and the Gene Expression database of Normal and Tumor tissues 2 (GENT2) [24] were used to examine the differences in *SHANK2* expression between the kidney cancer and the adjacent normal tissues. All datasets containing cancer versus normal at the mRNA expression levels were retrieved from the databases, and the relevant information was extracted. In total, 19 kidney gene expression datasets, comprising 1509 RCC and 414 adjacent normal tissues, were included in the analysis. The pooled standardised mean differences and 95% confidence intervals (CIs) were used to determine the differential gene expression between RCC and adjacent normal tissues. The Review Manager 5.4.1 (Cochrane Collaboration, London, UK) was used to evaluate heterogeneity among studies and perform the pooled analysis. Since substantial heterogeneity is present (Q test *p* < 0.05 and *I*^2^ > 50%) among included studies, random-effects model was used to calculate the summary statistics. In addition, correlations of the gene expression levels with the tissue type, stage, and survival of patients with RCC were assessed using data from TCGA KICH, KIRC, and KIRP datasets. The RNA-sequencing (RNA-Seq by expectation-maximization) data and corresponding clinical information were obtained from the Genomic Data Commons Data Portal, and the associations of *SHANK2* expression levels with tumour stage and survival of patients with RCC were assessed using Spearman’s correlation analysis and Kaplan–Meier survival curves, respectively.

### 2.4. Statistical Analyses

A chi-squared test was performed to examine differences in demographic distributions between the patients with RCC and healthy controls. Logistic regression analysis was used to evaluate the associations between genetic variants and the risk of RCC. The associations of genetic variants with the survival of patients with RCC were assessed using Cox proportional hazards regression analysis. All statistical analyses were performed using the Statistical Package for the Social Sciences version 19.0.0 (IBM, Armonk, NY, USA) with *p* < 0.05 as a significance threshold. False discovery rates (*q* values) were computed for multiple testing corrections [25].

## 3. Results

The baseline characteristics of the 312 patients with RCC and 318 healthy controls are summarised in Table 1. There were no significant differences between the patients and controls regarding the age and sex (*p* > 0.05). Ever alcohol consumption was related to reduced RCC risk, whereas hypertension and diabetes were related to increased risk of RCC (*p* < 0.001). Moreover, 34 (10.9%) patients died during a median follow-up time of 90.0 months.

The associations between the SHANK family gene polymorphisms and the risks of RCC are presented in Table S1. Among the 161 SNPs, *SHANK2* rs10792565 showed a significant association with the risk of RCC after adjusting for multiple testing. The odds of developing RCC were estimated to be increased by 79% with each copy of the rs10792565 minor T allele (odds ratio (OR) = 1.79, 95% CI = 1.32–2.44, *p* = 1.96 × 10^−4^, *q* = 0.030; Table 2). After adjusting for potential variables listed in Table 1 (age, gender, body mass index, cigarette smoking status, alcohol consumption, and histories of hypertension and diabetes), multivariate analysis showed that the *SHANK2* rs10792565 T allele remained significantly correlated with the risk of developing RCC (adjusted OR = 1.75, 95% CI = 1.25–2.44, *p* = 0.001; Table 2). Furthermore, preliminary results revealed that nine SNPs tended to correlate with overall survival of patients with RCC, but none of them reached the significance level after adjustment for multiple comparisons (*q* > 0.05; Table S1).

**Figure 1:**
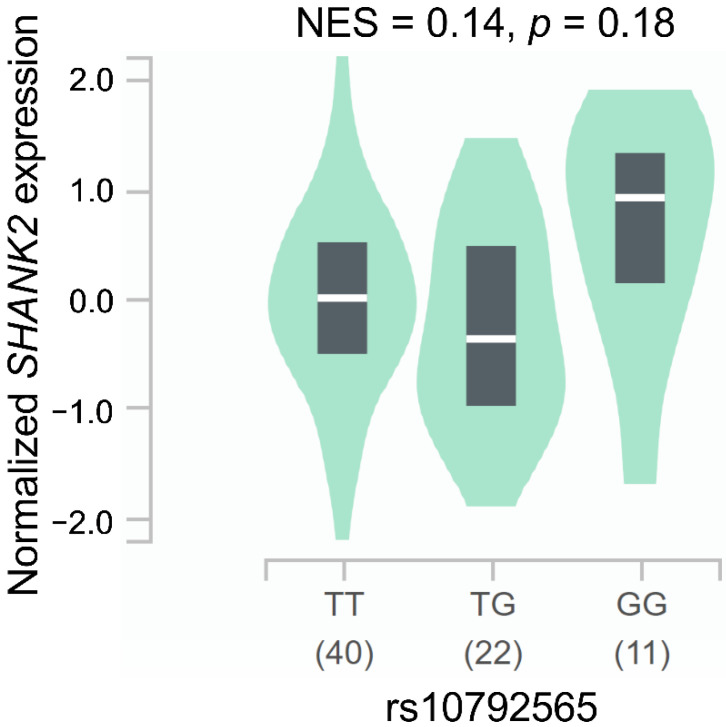
Association of *SHANK2* expression levels with the rs10792565 genotypes in normal kidney cortex tissue based on the Genotype Tissue Expression data. Values in brackets represent the number of patients. NES, normalized effect size.

Next, function prediction was performed for *SHANK2* rs10792565 and its proxy SNPs that are in high linkage disequilibrium using bioinformatics tools. According to the HaploReg database, rs10792565 and its linked (*r*^2^ > 0.8) SNP rs10897838 are in a protein-binding region of SET domain bifurcated histone lysine methyltransferase 1; alter the doublesex- and mab-3-related transcription factor 5 and oestrogen receptor-α regulatory motifs; and have been identified as an expression quantitative trait locus for *SHANK2* in three studies (Table 3). However, the rs10792565 risk allele T showed only a correlation trend with lower *SHANK2* mRNA expression levels in normal human kidney cortex tissues in the GTEx database, likely due to a small sample size (*n* = 73; Figure 1).

**Figure 2:**
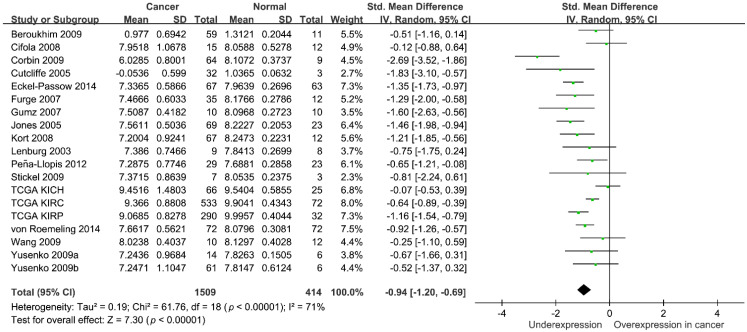
Pooled analysis of *SHANK2* expression levels between cancer and normal tissues in 19 independent kidney cancer studies. Std., standardized; SD, standard deviation; IV, inverse variance; CI, confidence interval; df, degrees of freedom.

**Figure 3:**
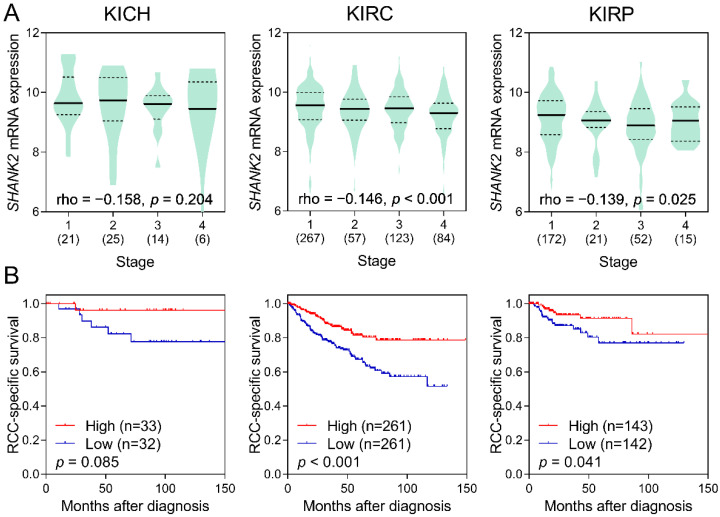
Correlation of *SHANK2* expression levels with renal cell carcinoma (RCC) progression. (**A**) Downregulation of *SHANK2* expression in advanced-stage RCCs, and (**B**) association of low *SHANK2* expression levels with a poor cancer-specific survival based on The Cancer Genome Atlas kidney chromophobe (KICH), kidney renal clear cell carcinoma (KIRC), and kidney renal papillary cell carcinoma (KIRP) data. Values in brackets represent the number of patients; rho, Spearman’s rank correlation coefficient.

To further evaluate the potential functions of *SHANK2* in RCC, we used publicly available kidney cancer datasets. A pooled analysis of 1509 kidney cancer tissues and 414 adjacent normal tissues from 19 independent studies demonstrated that *SHANK2* was downregulated in kidney cancers (*p* < 0.001; Figure 2). The relationship between *SHANK2* expression and the survival of patients with RCC was also analysed using three TCGA RCC datasets, KICH, KIRC, and KIRP. The expression of *SHANK2* was reduced in advanced-stage tumours in the KIRC and KIRP datasets (*p* < 0.001 and *p* = 0.025, respectively; Figure 3A), and low *SHANK2* expression levels were significantly associated with a poor survival of patients (*p* < 0.001 and *p* = 0.041, respectively; Figure 3B). Although there was a consistent trend in the KICH dataset, it did not reach statistical significance.

## 4. Discussion

By analysing SHANK family gene variants, we identified *SHANK2* rs10792565 as a novel variant associated with RCC risk in the present study. Furthermore, our pooled analysis of 19 independent studies revealed that the mRNA expression of *SHANK2* was downregulated in RCC compared with that in adjacent normal tissues, and lower expression levels of *SHANK2* were significantly associated with poorer survival of patients with RCC.

A functional analysis indicated that rs10792565, which is an intronic variant, might be potentially functional via its ability to alter regulatory binding motifs, and it has been described as an expression quantitative trait locus for *SHANK2* in human lymphoblastoid cells [26]. Although we only observed a tendency towards a correlation of the rs10792565 risk allele T with lower *SHANK2* expression in the human kidney cortex, it is possible that rs10792565 could also affect *SHANK2* mRNA splicing, folding, and protein translation. Additional experimental studies using site-directed mutagenesis and cycloheximide chase assay may be required to determine whether rs10792565 plays a role in the regulation of SHANK2 protein expression and stability.

A pooled analysis of public gene expression datasets showed downregulation of *SHANK2* in kidney cancer specimens, suggesting that this gene might play an important role during kidney carcinogenesis. SHANK2 is a member of the SHANK family consisting of three related multimodular scaffold proteins, which has mostly been studied in neuroscience to promote synapse formation. A recent study found that SHANK proteins also act as repressors of integrin activity by sequestering Ras family members, Rap1 and R-Ras, and consequently interfere with cell migration, spreading, and invasion [5]. It has been demonstrated that SHANK2 can serve as a master scaffold protein to recruit a type I metabotropic glutamate receptor (mGluR) and phospholipase C β3 into the same signalling complex, thereby influencing mGluR-induced intracellular calcium mobilisation [27]. Furthermore, *SHANK2* is frequently downregulated in neuroblastoma, and its decreased expression is associated with a poor survival of patients. On the other hand, overexpression of *SHANK2* in neuroblastoma cells results in increased cell differentiation and reduced cell growth following treatment with all-trans retinoic acid [28]. These findings suggest that *SHANK2* may play a tumour-suppressive role during cancer progression, which is in line with our observation in RCC. By contrast, *SHANK2* was found to be upregulated in oesophageal squamous cell carcinoma, and its high expression levels were associated with poor survival [29]. Some studies have reported that overexpression of *SHANK2* can suppress Hippo signalling by inhibiting large tumour suppressor kinase 1/2 mediated phosphorylation of Yes-associated protein 1, which results in uncontrolled cell proliferation [30]. Together, *SHANK2* plays important roles in cancers; however, its underlying mechanisms of action vary greatly depending on the cellular contexts. Therefore, further functional studies are warranted to validate our findings and elucidate how *SHANK2* rs10792565 is involved in RCC aetiology.

Although genetic studies from us and others have identified multiple RCC susceptibility genes, including caspase 9, AKT serine/threonine kinase 1, cyclin-dependent kinase inhibitor 2C [31], glutamate metabotropic receptors [15], hepatic leukaemia factor [32], and mitogen-activated protein kinase 10 [33], to our knowledge, this is the first study to link *SHANK2* to RCC. However, several limitations should be noted in the present study. The study cohort was recruited only from the Taiwanese population, and the sample size was relatively small. Therefore, further replication of the data should be confirmed in different ethnicities. Furthermore, although we integrated multiple bioinformatics data to decipher the possible mechanism underlying the observed association between *SHANK2* rs10792565 and an increased risk of RCC, additional functional experiments are needed to confirm the role of this SNP during RCC progression.

## 5. Conclusions

In conclusion, we found that *SHANK2* rs10792565 might contribute to the risk of RCC, and the expression level of *SHANK2* was correlated with the patient’s prognosis. Given the potential prognostic role of *SHANK2* in RCC, its genetic variants and expression levels may serve as novel prognostic biomarkers or for clinical decision-making for patients with RCC.

## Figures and Tables

**Table 1:** The clinical characteristics of the study population.

Characteristic	Cases (*n* = 312)	Controls (*n* = 318)	Age- and Sex-Adjusted OR (95% CI)
Age ≥ 58, *n* (%)	152 (48.7)	156 (49.1)	
Male, *n* (%)	209 (67.0)	210 (66.0)	
Body mass index ≥ 25, *n* (%)	138 (44.5)	123 (43.0)	1.07 (0.77–1.49)
Ever cigarette smoking, *n* (%)	114 (36.5)	105 (33.0)	1.18 (0.82–1.69)
Ever alcohol consumption, *n* (%)	76 (24.4)	135 (42.5)	0.39 (0.27–0.56)
Hypertension, *n* (%)	134 (43.1)	78 (24.9)	3.89 (2.25–6.65)
Diabetes, *n* (%)	62 (19.9)	20 (6.3)	2.49 (1.74–3.58)
Stage III–IV, *n* (%)	55 (18.6)		
Grade III–IV, *n* (%)	68 (24.8)		
Deaths ^a^, *n* (%)	34 (10.9)		

Abbreviations: OR, odds ratio; CI, confidence interval. ^a^ With median follow-up of 90.0 months.

**Table 2:** The association between *SHANK2* rs10792565 and RCC risk.

Genotype	Cases, *n* (%)	Controls, *n* (%)	OR (95% CI)	*p*	*q*	OR (95% CI) ^a^	*p* ^a^
GG	189 (61.0)	236 (74.2)	1.00			1.00	
GT	109 (35.2)	78 (24.5)	1.75 (1.23–2.47)	0.002		1.66 (1.13–2.44)	0.009
TT	12 (3.9)	4 (1.3)	3.75 (1.19–11.8)	0.024		3.92 (1.19–12.9)	0.025
Trend			1.79 (1.32–2.44)	1.96 × 10^−4^	0.030	1.75 (1.25–2.44)	0.001

Abbreviations: RCC, renal cell carcinoma; OR, odds ratio; CI, confidence interval. ^a^ ORs were adjusted for age, gender, body mass index, cigarette smoking status, alcohol consumption, and histories of hypertension and diabetes.

**Table 3:** Regulatory annotation of *SHANK2* rs10792565 and its linked proxy SNPs.

Chromosome	Position	SNP ID	LD (*r*^2^)	Reference Allele	Alternate Allele	AFR Frequency	ASN Frequency	EUR Frequency	Selected eQTL Hits	Proteins Bound	Motifs Changed
11	71227678	rs10792565	1	T	G	0.46	0.79	0.37	3 hits	SETDB1	
11	71236303	rs10897838	0.99	T	C	0.36	0.79	0.37	3 hits		DMRT5, ERalpha-a

Abbreviations: SNP, single-nucleotide polymorphism; LD, linkage disequilibrium; AFR, African; ASN, Asian; EUR, European; eQTL, expression quantitative trait loci.

## Data Availability

The data that support the findings of this study are available from the corresponding author upon reasonable request.

## Supplementary Materials

The following supporting information can be downloaded at: https://www.mdpi.com/article/10.3390/ijerph191912471/s1, Table S1. Genotyped SNPs and the *p* values of their associations with RCC risk and overall survival.
