# Supplementary material for: Genetic Analysis Implicates Dysregulation of SHANK2 in Renal Cell Carcinoma Progression
Source: Int J Environ Res Public Health. 2022 Sep 30;19(19):12471. doi: 10.3390/ijerph191912471 (PMC9566262; doi:10.3390/ijerph191912471)
Supplement: Supplementary file 1 [file ijerph-19-12471-s001.zip › ijerph-1904194-supplementary.pdf]

Table S1. Genotyped SNPs and the *P* values of their associations with RCC risk and overall survival

Gene	SNP ID	Chromosome	Position	MAF	HWE	Allele	Risk		Overall survival	
							P	*q*	*P*	*q*
SHANK1	rs35816100	19	50649692	0.079	0.056	T>C	0.312	0.892	0.136	0.532
SHANK1	rs35429431	19	50650175	0.196	0.387	T>C	0.136	0.695	0.074	0.466
SHANK1	rs117873682	19	50650766	0.089	1.000	C>A	0.610	0.897	0.474	0.578
SHANK1	rs11879379	19	50652265	0.111	0.164	C>T	0.522	0.897	0.237	0.532
SHANK1	rs889132	19	50655132	0.470	0.099	G>C	0.694	0.897	0.382	0.544
SHANK1	rs10404821	19	50657831	0.253	0.472	C>A	0.798	0.897	0.970	0.701
SHANK1	rs59500446	19	50661203	0.046	1.000	C>T	0.843	0.897	0.604	0.592
SHANK1	rs73596615	19	50662968	0.325	0.723	G>A	0.746	0.897	0.380	0.544
SHANK1	rs3893128	19	50672798	0.161	0.312	G>A	0.061	0.682	0.994	0.701
SHANK1	rs4802729	19	50675712	0.373	0.934	T>C	0.203	0.888	0.781	0.641
SHANK1	rs4802731	19	50678332	0.130	0.863	G>A	0.032	0.533	0.983	0.701
SHANK1	rs12460584	19	50682824	0.241	0.915	C>T	0.927	0.932	0.407	0.557
SHANK1	rs59312377	19	50683350	0.110	0.843	A>G	0.501	0.897	0.875	0.684
SHANK1	rs10406993	19	50689889	0.346	0.197	G>A	0.831	0.897	0.294	0.532
SHANK1	rs8103945	19	50690186	0.144	0.875	T>C	0.662	0.897	0.314	0.544
SHANK1	rs35259530	19	50691291	0.154	0.180	A>G	0.113	0.695	0.484	0.578
SHANK1	rs12459070	19	50692634	0.090	1.000	C>T	0.994	0.934	0.554	0.587
SHANK1	rs4802734	19	50696314	0.466	0.211	T>C	0.664	0.897	0.063	0.466
SHANK1	rs12461427	19	50702059	0.114	0.847	A>G	0.338	0.892	0.353	0.544
SHANK1	rs77136946	19	50704289	0.037	0.222	C>T	0.282	0.888	0.001	0.114
SHANK1	rs41275786	19	50704518	0.079	0.788	C>T	0.371	0.897	0.254	0.532
SHANK1	rs4801849	19	50707194	0.430	0.067	A>G	0.921	0.932	0.017	0.292
SHANK1	rs78855510	19	50707278	0.079	0.789	C>T	0.411	0.897	0.430	0.561

SHANK1	rs7246004	19	50713652	0.385	0.071	T>G	0.949	0.932	0.018	0.292
SHANK1	rs3745530	19	50714293	0.213	0.562	G>A	0.820	0.897	0.460	0.569
SHANK1	rs3826780	19	50717630	0.046	1.000	G>A	0.824	0.897	0.385	0.544
SHANK1	rs3810282	19	50717739	0.039	1.000	C>A	0.433	0.897	0.165	0.532
SHANK1	rs11084024	19	50725377	0.045	0.382	G>A	0.669	0.897	0.660	0.616
SHANK2	rs145975209	11	70483722	0.034	0.543	C>A	0.599	0.897	0.194	0.532
SHANK2	rs573795	11	70496312	0.332	0.861	G>A	0.065	0.682	0.269	0.532
SHANK2	rs11236504	11	70518554	0.182	0.794	C>T	0.040	0.605	0.050	0.466
SHANK2	rs12419914	11	70530319	0.395	0.371	C>T	0.265	0.888	0.557	0.587
SHANK2	rs11236569	11	70532867	0.348	0.607	G>A	0.810	0.897	0.151	0.532
SHANK2	rs76213548	11	70539179	0.055	0.042	G>A	0.525	0.897	0.922	0.686
SHANK2	rs73522161	11	70544357	0.228	0.739	G>A	0.777	0.897	0.008	0.292
SHANK2	rs11236600	11	70547213	0.369	0.358	A>G	0.749	0.897	0.689	0.616
SHANK2	rs138959027	11	70549823	0.034	0.539	T>G	0.572	0.897	0.750	0.622
SHANK2	rs80042712	11	70559047	0.090	0.471	C>T	0.570	0.897	0.018	0.292
SHANK2	rs12285102	11	70570756	0.216	0.818	T>C	0.870	0.906	0.007	0.292
SHANK2	rs77550108	11	70571334	0.092	1.000	T>C	0.991	0.934	0.011	0.292
SHANK2	rs11604385	11	70574867	0.034	1.000	C>T	0.713	0.897	0.930	0.686
SHANK2	rs948196	11	70578152	0.133	0.310	G>A	0.741	0.897	0.712	0.617
SHANK2	rs10899208	11	70578306	0.454	0.480	C>T	0.557	0.897	0.131	0.532
SHANK2	rs80178232	11	70583528	0.048	1.000	C>T	0.849	0.897	0.048	0.466
SHANK2	rs7127885	11	70585358	0.105	0.216	G>A	0.360	0.892	0.090	0.487
SHANK2	rs6592642	11	70592847	0.189	0.446	G>A	0.462	0.897	0.256	0.532
SHANK2	rs10793170	11	70593921	0.425	0.381	G>A	0.788	0.897	0.082	0.466
SHANK2	rs77778540	11	70595750	0.101	1.000	A>C	0.407	0.897	0.329	0.544
SHANK2	rs4980621	11	70597406	0.112	1.000	C>T	0.834	0.897	0.700	0.616

SHANK2	rs948193	11	70598341	0.364	0.737	G>A	0.800	0.897	0.068	0.466
SHANK2	rs146305493	11	70600507	0.047	1.000	G>A	0.940	0.932	0.153	0.532
SHANK2	rs4980625	11	70605947	0.353	1.000	G>T	0.143	0.695	0.942	0.690
SHANK2	rs9665887	11	70610446	0.054	0.249	T>C	0.854	0.897	0.705	0.616
SHANK2	rs61195439	11	70614305	0.256	0.541	T>C	0.558	0.897	0.525	0.587
SHANK2	rs72946910	11	70622083	0.304	0.712	G>A	0.719	0.897	0.235	0.532
SHANK2	rs79654220	11	70625394	0.076	0.787	T>C	0.329	0.892	0.350	0.544
SHANK2	rs558830	11	70629412	0.325	0.859	T>C	0.852	0.897	0.358	0.544
SHANK2	rs77483217	11	70640604	0.033	1.000	C>T	0.224	0.888	0.461	0.569
SHANK2	rs12277496	11	70644848	0.268	0.113	A>C	0.299	0.892	0.221	0.532
SHANK2	rs563532	11	70646303	0.449	1.000	C>G	0.268	0.888	0.614	0.592
SHANK2	rs471859	11	70647992	0.212	0.815	T>C	0.078	0.682	0.523	0.587
SHANK2	rs117035625	11	70657245	0.039	1.000	A>G	0.610	0.897	0.275	0.532
SHANK2	rs525304	11	70660101	0.289	0.777	G>T	0.255	0.888	0.633	0.604
SHANK2	rs61233494	11	70668305	0.110	0.843	A>G	0.451	0.897	0.747	0.622
SHANK2	rs10899375	11	70683045	0.280	0.211	T>C	0.079	0.682	0.422	0.561
SHANK2	rs17161065	11	70688406	0.084	0.305	T>C	0.170	0.776	0.192	0.532
SHANK2	rs79812988	11	70700098	0.061	0.161	A>G	0.014	0.389	0.608	0.592
SHANK2	rs12421506	11	70710009	0.265	0.366	C>T	0.119	0.695	0.287	0.532
SHANK2	rs118165285	11	70721599	0.031	0.127	T>G	0.711	0.897	0.898	0.684
SHANK2	rs74236607	11	70731218	0.082	0.793	C>T	0.761	0.897	0.164	0.532
SHANK2	rs118132458	11	70740364	0.068	0.760	C>A	0.553	0.897	0.867	0.684
SHANK2	rs550832	11	70745633	0.309	0.855	G>A	0.452	0.897	0.514	0.587
SHANK2	rs149405382	11	70786000	0.045	0.035	G>A	0.740	0.897	0.589	0.592
SHANK2	rs75332771	11	70801971	0.047	0.167	A>C	0.636	0.897	0.695	0.616
SHANK2	rs1992674	11	70802301	0.225	0.265	T>C	0.361	0.892	0.581	0.592

SHANK2	rs76079118	11	70809822	0.037	0.054	G>T	0.836	0.897	0.274	0.532
SHANK2	rs3017493	11	70819416	0.060	1.000	A>G	0.278	0.888	0.279	0.532
SHANK2	rs11237599	11	70820660	0.088	0.085	C>T	0.996	0.934	0.540	0.587
SHANK2	rs12295625	11	70825755	0.043	1.000	G>A	0.738	0.897	0.538	0.587
SHANK2	rs144847184	11	70830864	0.032	0.490	A>G	0.307	0.892	0.374	0.544
SHANK2	rs2921340	11	70838719	0.414	1.000	C>T	0.281	0.888	0.558	0.587
SHANK2	rs3017479	11	70855025	0.122	0.465	T>C	0.539	0.897	0.928	0.686
SHANK2	rs10899616	11	70879130	0.310	0.203	G>A	0.718	0.897	0.026	0.369
SHANK2	rs530497	11	70889612	0.109	1.000	G>A	0.314	0.892	0.250	0.532
SHANK2	rs34944804	11	70901936	0.141	0.872	A>C	0.981	0.934	0.731	0.622
SHANK2	rs3020095	11	70903916	0.143	0.340	C>T	0.639	0.897	0.355	0.544
SHANK2	rs79477765	11	70907773	0.119	0.713	C>T	0.279	0.888	0.203	0.532
SHANK2	rs7113981	11	70925728	0.271	0.767	G>A	0.661	0.897	0.835	0.672
SHANK2	rs76274790	11	70925838	0.036	1.000	C>G	0.080	0.682	0.219	0.532
SHANK2	rs4074929	11	70927426	0.243	0.833	C>T	0.357	0.892	0.271	0.532
SHANK2	rs78885129	11	70954765	0.106	0.413	G>T	0.471	0.897	0.214	0.532
SHANK2	rs4262749	11	71100226	0.274	1.000	C>A	0.741	0.897	0.615	0.592
SHANK2	rs117467179	11	71100415	0.052	0.413	A>G	0.129	0.695	0.080	0.466
SHANK2	rs73521173	11	71118855	0.032	0.027	C>T	0.954	0.932	0.064	0.466
SHANK2	rs137945425	11	71123086	0.038	0.618	C>G	0.020	0.423	0.403	0.557
SHANK2	rs117253486	11	71129376	0.090	1.000	A>C	0.556	0.897	0.888	0.684
SHANK2	rs11232163	11	71129751	0.352	0.608	C>T	0.137	0.695	0.152	0.532
SHANK2	rs10792488	11	71137802	0.486	0.311	G>A	0.118	0.695	0.168	0.532
SHANK2	rs9888203	11	71149721	0.144	0.431	G>T	0.855	0.897	0.785	0.641
SHANK2	rs7941128	11	71177012	0.480	0.755	A>C	0.238	0.888	0.892	0.684
SHANK2	rs11232477	11	71192792	0.118	0.710	A>G	0.475	0.897	0.876	0.684

SHANK2	rs58984468	11	71194944	0.107	1.000	T>C	0.542	0.897	0.993	0.701
SHANK2	rs11232512	11	71200067	0.062	0.091	C>T	0.270	0.888	0.591	0.592
SHANK2	rs74748300	11	71200444	0.132	1.000	G>A	0.637	0.897	0.439	0.566
SHANK2	rs55712817	11	71201995	0.109	0.843	T>C	0.382	0.897	0.993	0.701
SHANK2	rs147523086	11	71202212	0.042	0.621	G>A	0.013	0.389	0.565	0.589
SHANK2	rs59417145	11	71207990	0.144	1.000	G>A	0.965	0.934	0.295	0.532
SHANK2	rs11232573	11	71208647	0.343	0.434	G>A	0.013	0.389	0.170	0.532
SHANK2	rs11232576	11	71209545	0.146	1.000	A>G	0.796	0.897	0.272	0.532
SHANK2	rs11232591	11	71213183	0.145	1.000	G>A	0.901	0.932	0.272	0.532
SHANK2	rs7929059	11	71214813	0.361	0.737	A>G	0.382	0.897	0.428	0.561
SHANK2	rs1470162	11	71215246	0.315	0.788	G>A	0.103	0.695	0.927	0.686
SHANK2	rs10792551	11	71216017	0.402	0.936	A>C	0.798	0.897	0.692	0.616
SHANK2	rs141482340	11	71220704	0.030	1.000	T>A	0.821	0.897	0.373	0.544
SHANK2	rs147674252	11	71223556	0.051	0.684	T>C	0.774	0.897	0.700	0.616
SHANK2	rs74937165	11	71225150	0.056	1.000	A>G	0.122	0.695	0.574	0.592
SHANK2	rs10792565	11	71227678	0.174	0.685	G>T	0.000	0.030	0.742	0.622
SHANK2	rs10897855	11	71245451	0.494	0.534	T>G	0.574	0.897	0.370	0.544
SHANK2	rs143643492	11	71248057	0.052	1.000	C>A	0.706	0.897	0.106	0.532
SHANK2	rs1372108	11	71254030	0.036	0.208	G>A	0.083	0.682	0.461	0.569
SHANK2	rs7130526	11	71255627	0.443	0.097	G>A	0.928	0.932	0.140	0.532
SHANK2	rs10751061	11	71256388	0.486	0.877	G>C	0.450	0.897	0.384	0.544
SHANK2	rs2119509	11	71258636	0.160	0.563	A>G	0.130	0.695	0.822	0.667
SHANK3	rs6010044	22	50663510	0.398	0.167	A>C	0.062	0.682	0.723	0.622
SHANK3	rs9616906	22	50666252	0.059	0.720	G>A	0.525	0.897	0.069	0.466
SHANK3	rs9616907	22	50668551	0.060	0.723	G>C	0.444	0.897	0.068	0.466
SHANK3	rs75265108	22	50671289	0.045	0.634	G>A	0.830	0.897	0.071	0.466

SHANK3	rs75347843	22	50673933	0.404	0.684	G>A	0.015	0.389	0.736	0.622
SHANK3	rs76982449	22	50676230	0.101	0.667	G>A	0.337	0.892	0.057	0.466
SHANK3	rs12484572	22	50677435	0.400	0.516	G>A	0.011	0.389	0.971	0.701
SHANK3	rs9616915	22	50679152	0.067	1.000	T>C	0.359	0.892	0.336	0.544
SHANK3	rs7286601	22	50682988	0.061	1.000	T>G	0.278	0.888	0.187	0.532
SHANK3	rs9616934	22	50684165	0.173	0.585	C>A	0.229	0.888	0.515	0.587
SHANK3	rs139076185	22	50684673	0.062	1.000	G>A	0.537	0.897	0.915	0.686
SHANK3	rs8138473	22	50696460	0.305	0.199	C>T	0.509	0.897	0.444	0.566
SHANK3	rs9968024	22	50706303	0.374	0.319	T>C	0.156	0.735	0.683	0.616
SHANK3	rs147487861	22	50709307	0.032	0.490	C>T	0.086	0.682	0.187	0.532
SHANK3	rs1024374	22	50710892	0.072	0.566	G>C	0.637	0.897	0.222	0.532
SHANK3	rs73174419	22	50713256	0.052	0.248	G>A	0.956	0.932	0.480	0.578
SHANK3	rs9616948	22	50714126	0.220	0.428	A>G	0.639	0.897	0.662	0.616
SHANK3	rs6009953	22	50716723	0.414	0.689	C>T	0.427	0.897	0.668	0.616
SHANK3	rs12628091	22	50717305	0.111	0.553	T>C	0.422	0.897	0.327	0.544
SHANK3	rs76239491	22	50717681	0.163	0.886	G>A	0.075	0.682	0.614	0.592
SHANK3	rs6010062	22	50718304	0.455	0.238	A>G	0.835	0.897	0.238	0.532
SHANK3	rs73174424	22	50719055	0.043	1.000	C>T	0.253	0.888	0.552	0.587
SHANK3	rs6010065	22	50719589	0.452	0.159	C>G	0.979	0.934	0.252	0.532
SHANK3	rs12158487	22	50722853	0.112	0.555	C>A	0.490	0.897	0.306	0.543
SHANK3	rs10451	22	50723631	0.430	0.937	G>A	0.569	0.897	0.425	0.561
SHANK3	rs715586	22	50724710	0.113	0.439	C>T	0.550	0.897	0.290	0.532
SHANK3	rs192700691	22	50725482	0.054	0.250	C>T	0.551	0.897	0.199	0.532
SHANK3	rs6009957	22	50725859	0.438	1.000	T>C	0.621	0.897	0.516	0.587
SHANK3	rs8137951	22	50727236	0.415	0.749	G>A	0.357	0.892	0.531	0.587
SHANK3	rs2301584	22	50733069	0.154	0.368	G>A	0.345	0.892	0.509	0.587

SHANK3	rs41281537	22	50733239	0.159	0.663	G>A	0.092	0.691	0.876	0.684
SHANK3	rs756638	22	50733265	0.225	0.312	G>A	0.024	0.456	0.323	0.544
SHANK3	rs5770825	22	50735903	0.135	0.505	G>C	0.139	0.695	0.236	0.532
SHANK3	rs3810648	22	50737198	0.053	0.248	A>G	0.470	0.897	0.210	0.532
SHANK3	rs77216309	22	50738594	0.036	1.000	C>T	0.955	0.932	0.890	0.684
SHARPIN	rs2070688	8	144097007	0.258	0.032	T>C	0.281	0.888	0.293	0.532
SHARPIN	rs77359862	8	144099379	0.039	0.267	G>A	0.949	0.932	0.388	0.544
SHARPIN	rs117299156	8	144100763	0.041	0.296	T>C	0.627	0.897	0.704	0.616

Abbreviations: SNP, single nucleotide polymorphism; RCC, renal cell carcinoma; MAF, minor alleles frequency; HWE, Hardy-Weinberg equilibrium.
